# Elevated temperature increases meiotic crossover frequency via the interfering (Type I) pathway in *Arabidopsis thaliana*

**DOI:** 10.1371/journal.pgen.1007384

**Published:** 2018-05-17

**Authors:** Jennifer L. Modliszewski, Hongkuan Wang, Ashley R. Albright, Scott M. Lewis, Alexander R. Bennett, Jiyue Huang, Hong Ma, Yingxiang Wang, Gregory P. Copenhaver

**Affiliations:** 1 Department of Biology and the Integrative Program for Biological and Genome Sciences, University of North Carolina at Chapel Hill, Chapel Hill, North Carolina, United States of America; 2 State Key Laboratory of Genetic Engineering and Collaborative Innovation Center of Genetics and Development, Ministry of Education Key Laboratory of Biodiversity Sciences and Ecological Engineering, Institute of Plant Biology, School of Life Sciences, Fudan University, Shanghai, China; 3 Lineberger Comprehensive Cancer Center, University of North Carolina School of Medicine, Chapel Hill, North Carolina, United States of America; Stanford University School of Medicine, UNITED STATES

## Abstract

For most eukaryotes, sexual reproduction is a fundamental process that requires meiosis. In turn, meiosis typically depends on a reciprocal exchange of DNA between each pair of homologous chromosomes, known as a crossover (CO), to ensure proper chromosome segregation. The frequency and distribution of COs are regulated by intrinsic and extrinsic environmental factors, but much more is known about the molecular mechanisms governing the former compared to the latter. Here we show that elevated temperature induces meiotic hyper-recombination in *Arabidopsis thaliana* and we use genetic analysis with mutants in different recombination pathways to demonstrate that the extra COs are derived from the major Type I interference sensitive pathway. We also show that heat-induced COs are not the result of an increase in DNA double-strand breaks and that the hyper-recombinant phenotype is likely specific to thermal stress rather than a more generalized stress response. Taken together, these findings provide initial mechanistic insight into how environmental cues modulate plant meiotic recombination and may also offer practical applications.

## Author summary

Meiosis is the cell division used by sexually reproducing species to produce sperm and egg cells. During meiosis, programmed Double Strand Breaks (DSBs) occur on each chromosome, which allows DNA to be exchanged between chromosome pairs, resulting in crossovers (COs). COs are necessary to ensure faithful chromosome segregation during meiosis, and thus fertility, but are also an important source of genetic variation. As such, CO formation is tightly regulated. Despite this, CO frequency can be altered by external factors, such as temperature. In *Arabidopsis thaliana*, COs are formed through two pathways: interference-sensitive (Type I) and interference-insensitive (Type II). An increase in temperature results in an increase in CO frequency. Using a pollen based assay, we show that COs are formed in the Type I pathway, which accounts for approximately 85% of the COs in Arabidopsis. To investigate whether temperature-dependent COs are the result of additional DSBs, we used immunological staining to examine protein foci, which mark the sites of DSBs. We discovered that temperature likely increases CO frequency by shifting alternative repair outcomes, called non-crossovers, to favor additional COs, rather than by increasing DSBs. Lastly, we found that temperature is not a general stress response, as plants subject to salt stress did not exhibit an increase in CO frequency. Our results may prove valuable in aiding plant breeding by enhancing our ability to rapidly introgress suites of elite traits from wild-plants into their crop relatives, a method that is particularly attractive as it does not require genetic modifications.

## Introduction

Sexually reproducing species use a specialized form of cell division known as meiosis to create haploid gametes from diploid progenitor cells (or a similar genomic reduction in polyploids) [1]. A defining feature of meiosis is the exchange, or crossing-over, of DNA between homologous chromosomes. This exchange results in novel allelic combinations not present in either set of parental chromosomes. In most organisms, crossovers (COs) are also critical for stabilizing homologous chromosome pairing, thus ensuring proper segregation of homologous chromosomes during meiosis. In the absence of COs, chromosomes segregate randomly, resulting in imbalances in chromosome numbers in the gametes, and aneuploidy in progeny. Aneuploidy may affect the fertility of the organism and the viability and fertility of its offspring. Perhaps not surprisingly, the frequency and distribution of COs in the genome are genetically regulated.

CO formation is initiated by the creation of Spo11-induced DNA double-strand breaks (DSBs) [2]. In Arabidopsis meioses, approximately 200 DSBs are formed in each meiocyte, but only about 10 are repaired to form COs [3–6]. The remaining DSBs are repaired as non-crossovers (NCOs), presumably through the Synthesis-Dependent Strand Annealing (SDSA) pathway, or conceivably via sister chromatid repair [7]. Perturbations in DSB frequency do not concomitantly alter CO frequency, indicating that, at least to some extent, CO frequency is under homeostatic control [8]. Despite this, CO number and position can be modulated by external factors such as nutrient availability, exposure to environmental toxins, stress, and temperature [9,10]. How these cues are sensed by meiocytes and how they alter processes such as DSB formation and CO regulation are not known.

The ability of temperature to influence CO numbers was noted only four years after the first genetic map was constructed [11,12]. Many plants, including *Hordeum vulgare*, *Vicia faba*, *Hyacinthus orientalis*, and *Arabidopsis thaliana* have elevated CO frequencies at moderately higher temperatures [9,10], but the molecular mechanisms that mediate CO frequency changes have not been identified. Possible mechanisms for temperature-induced changes in CO frequency include direct effects of temperature on proteins that execute the steps in meiotic recombination, alteration of chromosome axis or synaptonemal complex structure, modulation of chromatin states, and changes in epigenetic regulations, such as DNA methylation.

In Arabidopsis, COs are formed through the Type I and Type II pathways [13]. The majority (~85%) of COs are derived from the Type I pathway in Arabidopsis and are sensitive to the placement of adjacent crossovers (interference sensitive); Type II COs make up most of the remainder of COs and are not sensitive to the placement of adjacent COs [14,15]. Many of the characterized hyper-recombinant mutants in Arabidopsis, including *fancm*, *figl*, *top3α*, and *recq4A/B*, operate through a shift in designation of recombination intermediates from NCOs to COs in the Type II pathway, resulting in drastic increases in CO frequency [16–18], much like those seen at 28°C. It is not known if temperature-induced COs in Arabidopsis are formed through the Type I, Type II, or both pathways.

To build an understanding of the molecular mechanisms that govern thermal control of CO frequency in plants, we employed a pollen-based visual assay for recombination in *Arabidopsis thaliana* to demonstrate that thermal stress-induced COs are generated by the interference sensitive Type I pathway and that they act additively with Type II pathway perturbations resulting in an enhanced hyper-recombination phenotype. We also show that this response is temperature-specific rather than a general stress response and that the extra COs occur without increasing the number of meiotic double-strand breaks. We would also like to note that in the process of submitting our manuscript, Lloyd et al., have published an early online manuscript that also shows in increase in Type I COs at elevated temperature in Arabidopsis [19].

## Results

### Temperature-dependent COs are derived from the Type I pathway

We had previously used a pollen-based, fluorescent tagged line (FTL) system to demonstrate that Arabidopsis grown at standard 20°C conditions experiences elevated CO frequencies when shifted to 28°C [20]. In brief, the FTL system employs pairs of transgenes encoding fluorescent proteins at defined genetic intervals. These transgenes are expressed under the pollen-specific, post-meiotic promoter, *LAT52* [21]. These markers are deployed in a *qrt1-2* background [22], which causes pollen from individual meioses to be shed as tetrads, allowing COs to be assayed visually by tracking the pattern of fluorescent protein expression in the tetrads.

**Fig 1:**
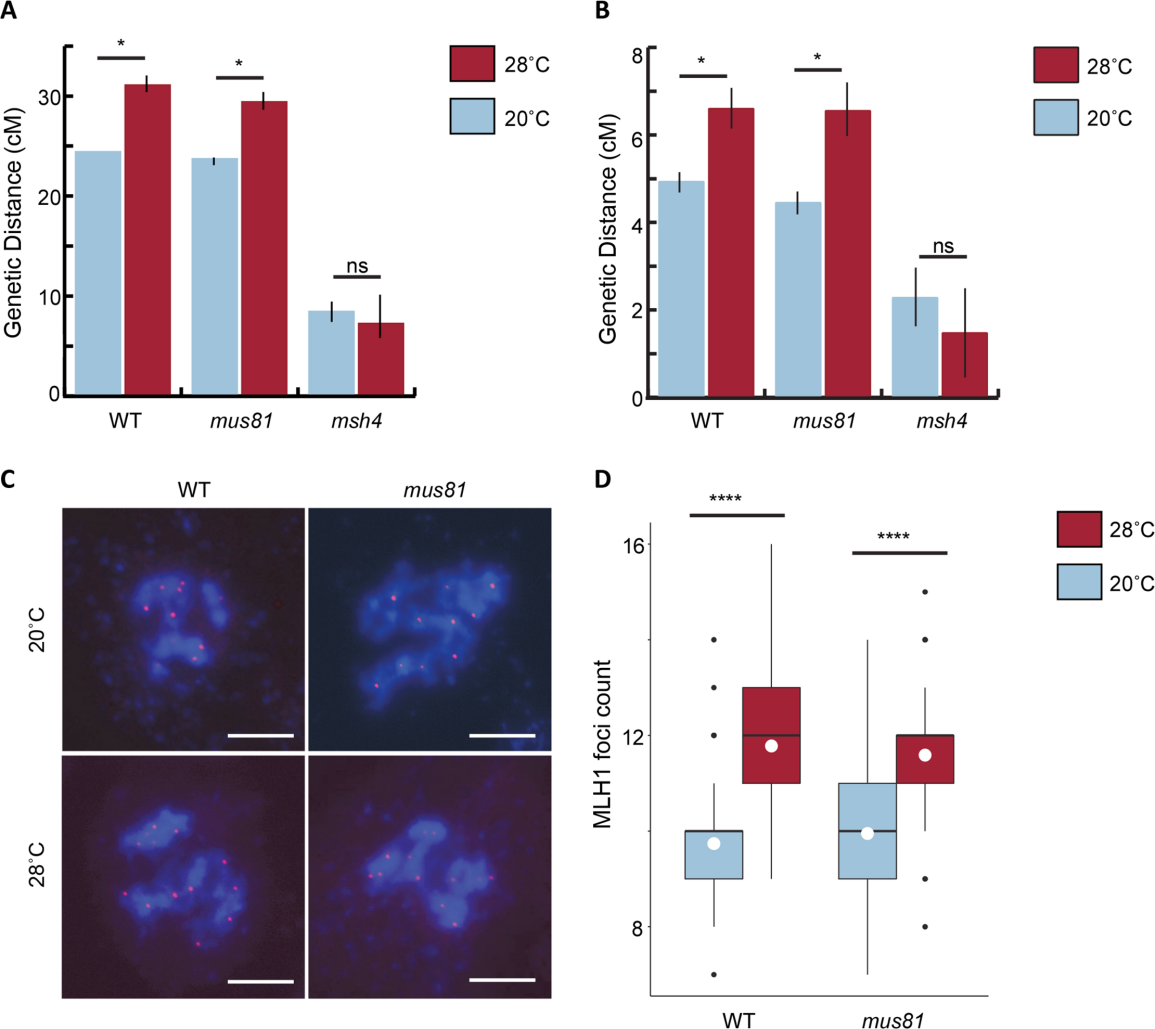
Temperature dependent modulation of meiotic recombination frequency occurs through the Type I CO pathway. (A, B) Genetic distances measured using FTLs in WT, *mus81*, and *msh4* plants at 20˚C and 28˚C in the I3a (A) and I1a (B) interval. Significantly different values between 20°C and 28°C at α = 0.05 are marked with an asterisk (*). (C, D) MLHI foci counting. (C) Pollen mother cells containing chromosomes (DAPI, blue) and MLHI foci (red). (D) Boxplot of MLH1 foci counts, mean shown as white circle; **** indicates p ≤ 0.0001. Scale bars represent 5 μm.

To determine if temperature-dependent COs are derived from the Type I or Type II pathway, we analyzed mutant lines of *msh4* (At4g17380) and *mus81* (At4g30870), which disable the Type I and Type II pathways, respectively [14,15]. *MSH4* is a homolog of the bacterial mis-match repair gene *MutS* that has lost its MMR function in Arabidopsis [14]. MSH4 instead functions in the Type I meiotic recombination pathway along with other ZMM proteins such as ZIP4, MSH5, MER3, HEI10, SHOC1 and PTD [23,24], where it is thought to act early in meiotic prophase I to stabilize double-Holliday Junctions (dHJs) [25]. MUS81 is an endonuclease thought to play a role in the resolution of single- and double-HJs in Arabidopsis [26,27] that also mediates CO formation in the Type II pathway [15]. WT and *mus81* plants show an increase in meiotic CO frequency when grown at 28°C, whereas *msh4* plants do not (Fig 1A and 1B, S1 Table). As a separate method of confirming these results, we immunostained Arabidopsis male meiocytes at diakinesis using a MLH1 antibody. MLH1 is a MutL homolog that co-localizes with MLH3 in meiosis at sites that will become Type I COs [28]. As expected, in WT plants, a significant increase in MLH1 foci at 28°C (average = 11.8, n = 41) was observed relative to 20°C (average = 9.7, n = 57) (Fig 1C and 1D, S2 Table). In *mus81* plants, a similar trend in MLH1 foci at 28°C (average = 11.6, n = 34) was found relative to 20°C (average = 10, n = 60) (Fig 1C and 1D, S2 Table). We used a modified Alexander’s stain assay to assess pollen viability under our control and experimental conditions and observed a significant reduction (p-value = 3.110 × 10^−16^) in the number of viable pollen per anther at 28°C (average = 294.5, n = 28) compared to 20°C (average = 665.7, n = 22) (S3 Table). Together, these data indicate that the increase in COs seen at 28°C is driven by the Type I meiotic recombination pathway in Arabidopsis.

### Temperature-induced hyper-recombination does not compromise interference

CO interference occurs when one CO influences, typically reducing, the likelihood of a second nearby CO [29–31]. Since temperature-induced hyper-recombination acts through the Type I meiotic recombination pathway, which is sensitive to interference, rather than the interference insensitive Type II pathway, we asked whether thermal stress influences interference in Arabidopsis. To compare the strength of interference at normal and high temperatures, we utilized three linked FTL markers and measured whether COs between the first and second marker influenced the frequency of COs between the second and third marker. We calculated the genetic distance in the first interval in the presence of a CO in the adjacent interval (X_wi_) and without (X_wo_). In the case of no change in interference, an increase in both X_wi_ and X_wo_ will be seen, whereas an increase (or decrease) in interference will result in a change in X_wo_ without a corresponding change in X_wi_. Our results indicate a small but non-significant trend of increased interference in plants grown at 28°C (S1 Fig, S4 Table), suggesting that there is either no change in interference or that the effect is subtle.

### Temperature-dependent COs are additive with *fancm* anti-CO activity

**Fig 2:**
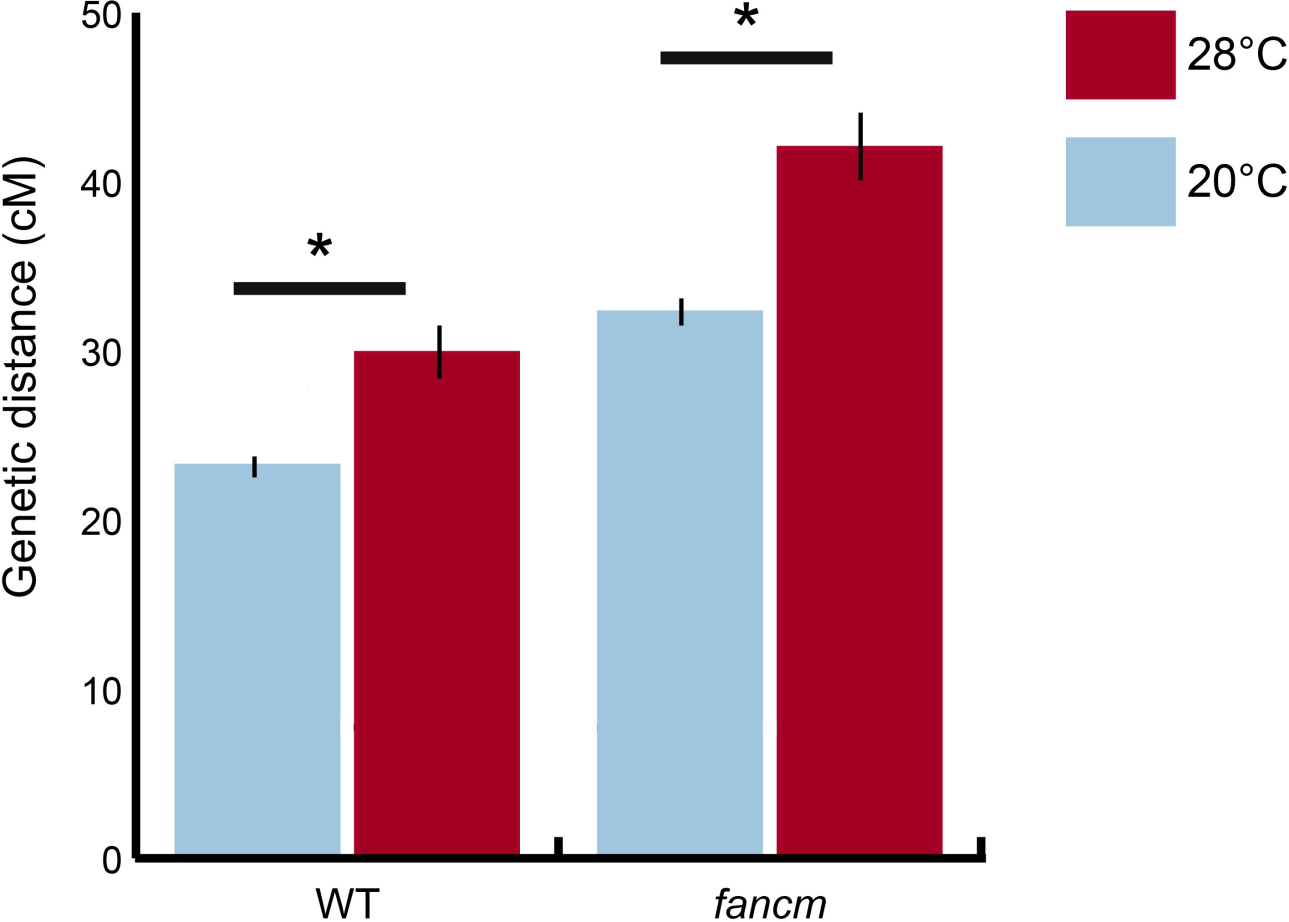
Temperature dependent modulation of meiotic CO frequency can work in conjunction with anti-CO factors. Genetic distances measured using FTLs in WT and *fancm* plants at 20°C and 28°C in the I3a interval. Significantly different values between 20°C and 28°C at α = 0.05 are marked with an asterisk (*).

Previous reports of hyper-recombination mutants have identified genes that antagonize CO formation in the Type II pathway, such as *FANCM*, *FIGL1*, TOP3α and *RECQ4A/B* [16–18]. The helicase FANCM is thought to process recombination intermediates into NCO products. In its absence, MUS81, but not the ZMM pathway, acts on these recombination intermediates and resolves them as COs, leading to an increase in CO frequency. TOP3α, RECQ4A/B, and FIGL1 also limit COs formation in the Type II pathway, although they do so independently of FANCM. To test if temperature-driven modulation of meiotic CO frequency can operate independently through the Type I pathway in absence of one of these anti-CO factors, we measured CO frequency in WT and *fancm* in a genetic interval on chromosome 3 (I3a). We observed an additive effective of temperature and *fancm* in lines grown at 28°C (Fig 2, S1 Table). Temperature-driven modulation of meiotic CO frequency through the Type I pathway thus appears to act autonomously of CO formation in the Type II pathway.

### Hyper-recombination is not a universal stress phenomenon

**Fig 3:**
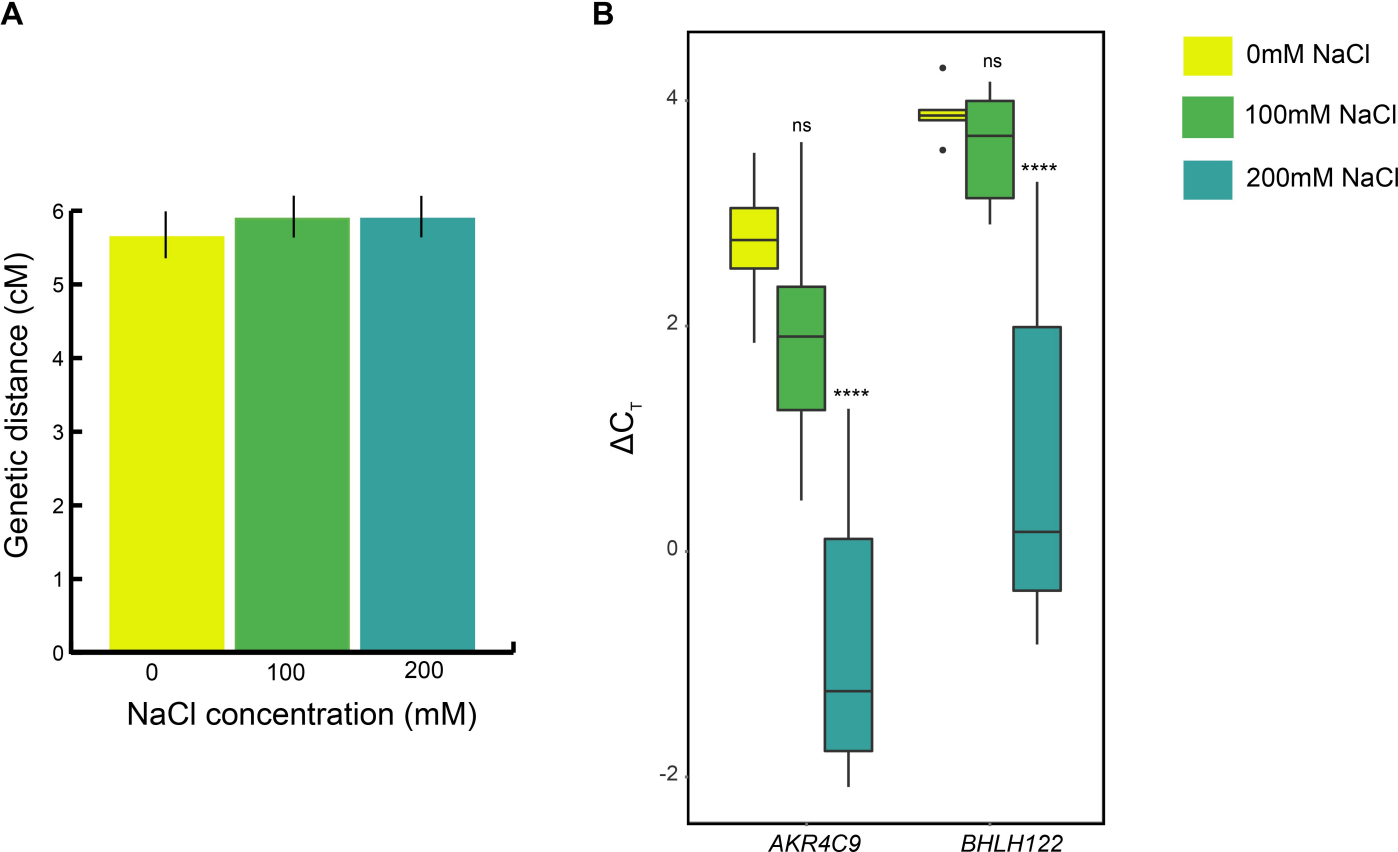
NaCl treatment does not induce changes in meiotic CO frequency. (A) Genetic distances measured in the I1a interval using FTL lines, showing SE; neither the 100 mM NaCl or 200 mM NaCl values are different from the control treatment. (B) ΔC_T_ values of *AKR4C9* and *BHLH122* for 0, 100, and 200 mM NaCl treatments, using *TUB4* as an endogenous control. Adjusted p-values are from Tukey’s honest significant difference test, and are indicated as follows: ns = p > 0.5, **** indicates p ≤ 0.0001.

In addition to temperature, other factors, such as nutrient availability, developmental stage, and chemicals have also been shown to modulate meiotic CO frequency in plants [9]. In order to test if other stresses induce an increase in CO frequency, we assayed CO frequency in WT plants grown under control and NaCl treatment conditions. No difference in CO frequency was observed between plants grown at 0, 100, and 200 mM NaCl (Fig 3A). To confirm that the NaCl treatment induced a stress response, we assayed the expression of the transcription factor *BHLH122*, an osmotic and drought stress biomarker [32], and the aldo/keto reductase family protein *AKR4C9*, an osmotic and salinity stress biomarker [33]. Although both 100 mM and 200 mM NaCl treated plants showed physiological signs of salinity stress (i.e., loss of turgor pressure), a significant increase in expression of both *BHLH122* and *AKR4C9* was only observed in the 200 mM treated plants. (Fig 3B, S5 Table). Plants treated with 100 mM NaCl showed no change in either *BHLH122* expression (fold change = 1.2, adjusted p-value = 0.837), or *AKR4C9* expression (fold change = 1.6, adjusted p-value = 0.315). Plants treated with 200 mM NaCl showed significant and marked stress-induced increases in expression of both *BHLH122* (fold change = 8.2 adjusted p-value = 4.4 × 10^−5^) and *AKR4C9* (fold change = 10.5, p-value = 1.81 × 10^−5^). Taken together, these results show that in Arabidopsis salt stress does not elicit the same meiotic hyper-recombination phenotype as thermal stress, suggesting that the latter is a specific rather than general stress response.

### Temperature-dependent COs are the result of a shift in CO designation through the Type I pathway, not an increase in DSBs

**Fig 4:**
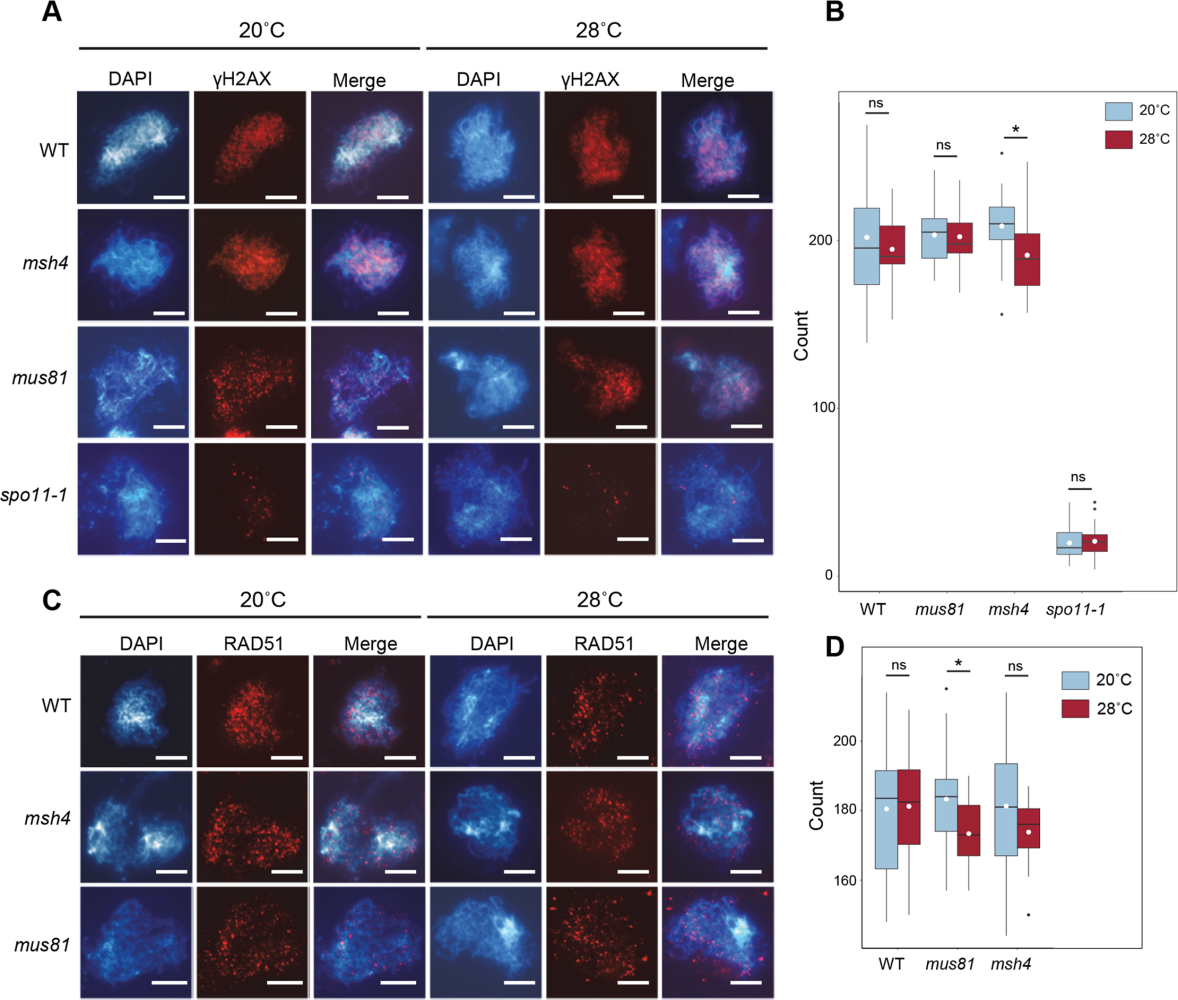
Temperature-dependent COs are not derived from an increase in DSBs. (A, B) γH2Ax foci counting. (A) Pollen mother cells containing chromosomes (DAPI, blue) and γH2Ax foci (red). (B) Boxplots of γH2Ax foci at 20˚C and 28˚C; mean shown as white circle. (C, D) RAD51 foci counting. (C) Pollen mother cells containing chromosomes (DAPI, blue) and RAD51 foci (red). (D) Boxplots of RAD51 foci at 20°C and 28°C; mean shown as white circle. p-values are indicated as follows: ns = p > 0.5, * = p ≤ 0.05, ** = p ≤ 0.01. Scale bars represent 5 μm.

CO frequency can be augmented by at least two mechanisms: an increase in the frequency of DSBs, which could concomitantly increase both NCOs and COs, or by shifting the ratio of NCO: COs to favor increased COs without a parallel increase in DSBs. To determine if temperature-induced COs are the result of an increase in DSBs rather than a shift in the NCO: CO ratio, we used immunostaining to count γH2AX and RAD51 foci in male meiocytes at zygotene of WT, *mus81*, and *msh4* plants. H2AX is a variant histone present in approximately 10% of nucleosomes that becomes phosphorylated (γH2AX) at serine 139 in response to DNA damage [34]. RAD51 is a homolog of the bacterial RecA protein that forms a nucleoprotein filament during recombination [35]. Both γH2AX and RAD51 foci mark the sites of meiotic DSBs in Arabidopsis [6,36]. In WT plants, the number of γH2AX foci (average = 201.8, n = 26) and RAD51 foci (average = 180.5, n = 24) at 20°C did not differ significantly from those at 28°C (average = 194.8, n = 16; average = 181.2, n = 14 respectively) (Fig 4, S6 Table, S7 Table). In *mus81* and *msh4* mutants, a trend towards fewer DSBs was observed at 28°C, although the results were neither highly significant nor seen in all cases (Fig 4, RAD51 in *mus81*, p-value = 0.042 and γH2AX in *msh4*, p-value = 0.035). In *spo11-1-1* mutants, no difference in γH2AX foci was seen in plants grown at 20°C (average = 19.8, n = 37) and 28°C (average = 20,8, n = 28) (Fig 4). As expected, the *spo11-1-1* mutants did exhibit a dramatic decrease in CO frequency relative to WT plants. These results lead us to reject the hypothesis that the extra COs observed at elevated temperatures in Arabidopsis are the result of an increase in DSBs.

## Discussion

Despite the necessity for COs to ensure proper chromosome segregation and evidence indicating that CO frequency is under homeostatic control, CO numbers can be altered by external factors, such as temperature. Here, we show that in Arabidopsis, these additional COs are formed through the interference-sensitive Type I pathway, and that the number of DSBs remains unchanged at 28˚C. This indicates that the increase in CO frequency is derived from a shift in the ratio of NCOs to COs. Although it is known that other factors can alter CO frequencies, we demonstrate here that in Arabidopsis an increase in CO frequency is not a ubiquitous response to stress.

Temperature-dependent modulation of meiotic CO frequency mimics the hyper-recombination phenotypes of Type II anti-CO factors mutants, such as *fancm*. As such, one may expect that temperature-dependent COs are derived from either the Type II pathway or both pathways. Surprisingly, we discovered that the temperature dependent COs are derived from the Type I pathway. These results contrast those seen in barley, where temperature-dependent COs appear to be derived from the Type II pathway and the distribution but not the frequency of Type I COs is changed [37]. At elevated temperatures, COs in barley redistributed along the length of the chromosomes, shifting from a terminalized to more medialized pattern. Our analysis did not provide the necessary resolution to detect a similar pattern in Arabidopsis, but chromosome field redistribution of COs has been observed in *met1* DNA methyltransferase mutants [38]. These observations raise the possibility that epigenetic mechanisms may play a role in regulating the frequency and distribution of COs in response to external cues.

Most sexually reproducing species generate an order of magnitude more meiotic DSBs compared to COs. In addition, most species have relatively few COs per chromosome, though there are interesting exceptions including several fungi, the SAR (Stramenopiles-Alveolates-Rhizaria Eukaryote) supergroup, and some insects [39–41]. At a molecular level, several factors work to limit the number of COs. FANCM and RECQ4A/B are both helicases that promote NCO formation by disassembling recombination intermediates that have formed D-loops and consequently directing them through the SDSA pathway, where they go on to form NCOs [16,18]. TOP3α works in conjunction with RECQ4A/B to maintain the recombination intermediates as NCOs, while FIGL inhibits homologous strand invasion, a necessary step for meiotic recombination [17]. Thus, it appears that despite an abundant pool of initiating events, and a default program that, if left unrestrained, will produce more COs, there is a common trend to limit COs. This problem may be particularly acute in polyploids, which must reduce CO numbers to avoid chromosome entanglements during segregation [39]. It is therefore intriguing that exposure to modest temperature increases, at least in laboratory settings, can cause plants to overcome these limiting programs and evoke a hyper-recombinant response. It would be interesting to explore whether polyploids block temperature-dependent hyper-recombination in addition to strengthening the limits on CO numbers. Our findings in this study and future elaboration of the molecular mechanisms used to increase CO frequency in response to external cues will enable such hypotheses to be tested.

DNA double-strand breaks occur in somatic cells as a result of temperature, osmotic, and oxidative stress, UV-radiation, and many other factors [42–45]. These breaks are repaired via non-homologous end-joining or somatic homologous recombination, effectively resulting in a relationship where external stressors increase somatic homologous recombination. In Arabidopsis, the increase in meiotic CO frequency in response to temperature is not a universal response to stress than can be replicated by other factors, such as salt stress. It should be noted that our assays do not exclude the possibility that salinity stress may result in a redistribution of COs along the chromosomes. It is also possible that even within Arabidopsis, the temperature response may be heterochiasmic. In barley, temperature only increases CO frequency in male meiosis, not female meiosis [46]. Furthermore, the distribution and frequency of COs differs between males and females in Arabidopsis [47,48]. Our pollen FTL system assays male meiosis only, thus it is possible that female meiosis may respond differently to elevated temperatures. Given the fluidity of the relationship between stress and CO frequency across many organisms, it seems that temperature-specific heterochiasmy should not be unexpected.

We selected 28°C as our thermal stress condition based on a range of elevated temperatures assayed in experiments used to originally detect the CO response [20]. Lloyd et al. observed an equally potent increase in COs at moderately cold (8°C- 13°C) temperatures as well [19]. All of our experiments have used the Columbia ecotype, which is adapted to a temperate climate. It would be interesting to test whether the thermal stress-induced CO phenotypes we observed also occur in ecotypes adapted to tropical climates, such as Cvi from the Cape Verde Islands [49], or cold-adapted ecotypes [50]. It is possible that temperate plants are particularly responsive to thermal stress cues and that plants from more extreme climates would have meiotic programs adapted to those conditions and would not be as responsive. For example, isolates of the fungus *Sordaria fimicola* from harsh micro-environments have higher recombination frequencies compared to those from mild micro-environments at the same collection site leading to the suggestion that adaptation to the harsh conditions had selected for increased COs [51], though in this case their response to changing conditions was not tested. Alternatively, plants adapted to more extreme climactic conditions may respond equally robustly but at different temperatures or “set points”.

While it is tempting to speculate that the temperature response observed here may be of adaptive value, it is equally possible that temperature dependent modulation of meiotic CO frequency is a function of physical factors in the cell, such as synaptonemal complex (SC) length. In barley, the increase in CO frequency observed in male meiosis is coupled with an increase in SC length, although the causal direction of the relationship is unclear [37]. In Arabidopsis, although it is unknown how SC length changes in response to temperature, SC length is longer in males, which also exhibit higher CO frequency [47,48]. It is also possible that the ability to modulate meiotic CO frequency in response to environmental cues may have initially been purely mechanistic in nature, but that the direction and magnitude of response may have subsequently been subject to selective pressure.

In addition to the potential for adaptive value, modulation of meiotic CO frequency via temperature is also of practical importance. Temperature-dependent modulation of meiotic CO frequency provides a means of aiding plant breeding without editing the genome or the necessity of working in mutant backgrounds; our observed reduction in pollen viability at high temperatures may require that more modest increases in temperature are utilized. Easily elevating CO frequency will reduce the number of F_2_ progeny necessary for classic genetic mapping of traits and will aid in disrupting persistent linkage blocks so that desirable traits can be isolated and bred into elite lines. Valuable genetic diversity, including disease resistance, remains locked in wild relatives and meiotic recombination limits the ability to introgress those traits into commercial relatives. Temperature treatment during reproductive stages may offer a simple and cost effective means to improve our ability to tap into these natural genetic resources.

## Materials and methods

### Plant lines

Seeds for mutant lines were obtained from the Arabidopsis Biological Resource Center; the following T-DNA lines were used: *MSH4* (At4g17380, SALK_136296), *MUS81* (At4g30870, SALK_107515), *FANCM* (At1g35530, SALK_120621). For *SPO11-1* (At3g13170) the *spo11-1-1* mutant [52] was used. FTL lines were generated as described previously [20]. Mutant lines are in the Columbia-0 ecotype background (CS60000) with the exception of the *spo11-1-1* line, which is derived from the Wassilewskija ecotype. FTL lines are in the Columbia-3 ecotype background (CS8846). DNA was extracted as described previously [3], and T-DNA lines were genotyped via PCR using primers and conditions provided in S8 Table.

### Plant growth and treatment conditions

Seeds were sown on Metromix-360 (Sun-Gro). Unless otherwise noted, plants were grown under 18 hour days at 20°C in a growth room for control conditions and at 28°C under 18 hours days in a Percival chamber for heat treatment conditions. For the heat treatment, flowering plants were placed in the 28°C chamber for five days and tetrads were counted on the fifth day. For the salt treatment, plants with dry soil were bottom watered until saturation with 0mM NaCl, 100mM NaCl, and 200mM NaCl. Tetrads were counted five days after treatment.

### Pollen tetrad assay

Crossover frequency was assayed via a visual fluorescent pollen transgene assay as described previously [20]. In the two color experiments (I1a and I3a intervals, S9 Table), tetrads were counted manually using a Nikon Eclipse E1000 epifluorescence microscope. In the three color experiment (I5cd interval, S9 Table), pollen grains were counted by first capturing an image of the entire slide for each sample on a Zeiss LSM 880 confocal laser scanning microscope, under the conditions provided in S1 File. Tetrads were then counted manually using Fiji [53] and a custom built program TetradAnalysis (https://github.com/jmodlis/TetradAnalysis). For all intervals, genetic distance was calculated using the Perkins equation [54], standard errors were calculated via Stahl Lab Online Tools (http://elizabethhousworth.com/StahlLabOnlineTools/), and p-values were calculated using R [55]. Interference was calculated following the method of Malkova et al [56]. The genetic distance in the I5c interval (FTL1963 and FTL1143, CFP and YFP respectively) measured in tetrads without a CO in the adjacent I5d interval (FTL1143 and FTL2450, YFP and dsRED2, respectively) was divided by the genetic distance in the I5c interval measured in tetrads with a CO in the adjacent I5d interval [57]. The difference in interference ratios at 20°C and 28°C was tested by calculating the p-value from the Z-score. The Z-score was calculated using the formula Z = |R_20_ –R_28_|/√(Var_R20_ + Var_R28_); the variance for each ratio was calculated as in van Kempen & van Vliet [58] and Stuart & Ord [59] with the exception that covariance was assumed to be zero.

### RT-PCR

RNA was collected from floral buds used in the salt treatment on the same day that tetrads were counted. RNA was extracted using the RNeasy Plant Mini Kit (Qiagen), followed by TURBO DNA-free DNase Treatment (Ambion). cDNA was generated using a ProtoScript II First Strand cDNA Synthesis Kit (New England Biolabs). One μg of cDNA was used in RT-PCR reactions, which were performed using the primers and conditions in S8 Table and the PowerUp SYBR Green Master Mix (Applied BioSystems) following the manufacturer’s instructions on a QuantStudio 6 Flex Real-Time PCR System (Applied Biosytems). Fold change (2^-ΔΔC^_T_) values for *BHLH122* (At1g51140) and *AKR4C9* (At2g37770) were calculated using the comparative C_T_ method of Schmittgen and Livak [60] with *TUB4* (At5g44340) as an endogenous control. To test for significant differences in gene expression between control and NaCl treatments for *BHLH122* and *AKR4C9*, ΔC_T_ values were used, where ΔC_T_ is the change in the expression of each gene relative to the endogenous control (e.g., ΔC_T(BHLH122)_—ΔC_T(TUB4)_). For each gene, an ANOVA was conducted to test for differences among ΔC_T_ means for all treatments, followed by a post-hoc Tukey’s honest significant difference test.

### Cytology

Immunofluorescence was performed as previously described by Wang et al., with minor modifications [39]. Unopened flower buds were fixed after 28°C and 20°C treatment for at least 24 hours in a manual climatic box (17600 Lux, 85% humidity, 16h light and 8h dark). The slides were incubated overnight with primary antibodies diluted 1:200 (RAD51, γH2AX, MLH1) in blocking buffer (goat serum, AR0009, Bosterbio) at 4°C and then at 37°C for 60 min with secondary antibody (1:1000, Goat anti-Rabbit IgG (H+L) Cross-Adsorbed Secondary Antibody, Alexa Fluor 555, catalog # A-21428, Thermo Fisher Scientific). Images were taken using a Zeiss Axio Imager A1 microscope and processed using Adobe Photoshop CS6. RAD51, γH2AX, and MLH1 foci were counted using Image Tool version 3.0 software (University of Texas Health Science Center, San Antonio, USA). The viability of pollen from control and temperature treated plants, grown and harvested as describe above, was assayed using the method described by Peterson et al [51]. Welch’s t-test was used to test for differences in means at 20°C and 28°C in R.

## Supporting information

S1 Fig Interference at 28°C. Genetic distances measured using the I5cd FTL interval in WT and *mus81*. Genetic distance measured in the I5c interval both with and without COs in the adjacent I5d interval. CFP, YFP and dsRED transgenes shown in blue, yellow, and red, respectively.
(TIF)

S1 Table Two color pollen count data from I3a and I1a intervals for WT, *mus81*, *msh4*, and *fancm* lines conducted in separate experiments. (XLSX)

S2 Table MLH1 foci data for WT, *msh4*, and *mus81* plants. (XLSX)

S3 Table Number of viable pollen grains per anther. (XLSX)

S4 Table Tetrad count data from the I5cd interval for WT and *mus81* plants grown at 20°C and 28°C. (XLSX)

S5 Table Raw qRTPCR data taken from QuantStudio 6 Flex Real-Time PCR system (Applied Biosystems), ΔCT, ΔΔCT, and 2-ΔΔCT calculations, and ANOVA results. (XLSX)

S6 Table RAD51 foci counts for WT, *msh4*, and *mus81* plants. (XLSX)

S7 Table γH2AX foci counts from WT, *msh4*, *mus81*, and *spo11-1-1* lines. (XLSX)

S8 Table PCR conditions and primers used in this study. (XLSX)

S9 Table Description of FTL intervals used in this study. (XLSX)

S1 File Microscope settings for the Zeiss LSM 880 confocal laser scanning microscope used in three color (I5cd) experiment. (PDF)
