# Supplementary figures and images for: Elevated temperature increases meiotic crossover frequency via the interfering (Type I) pathway in Arabidopsis thaliana
Source: PLoS Genet. 2018 May 17;14(5):e1007384. doi: 10.1371/journal.pgen.1007384 (PMC5976207; doi:10.1371/journal.pgen.1007384)

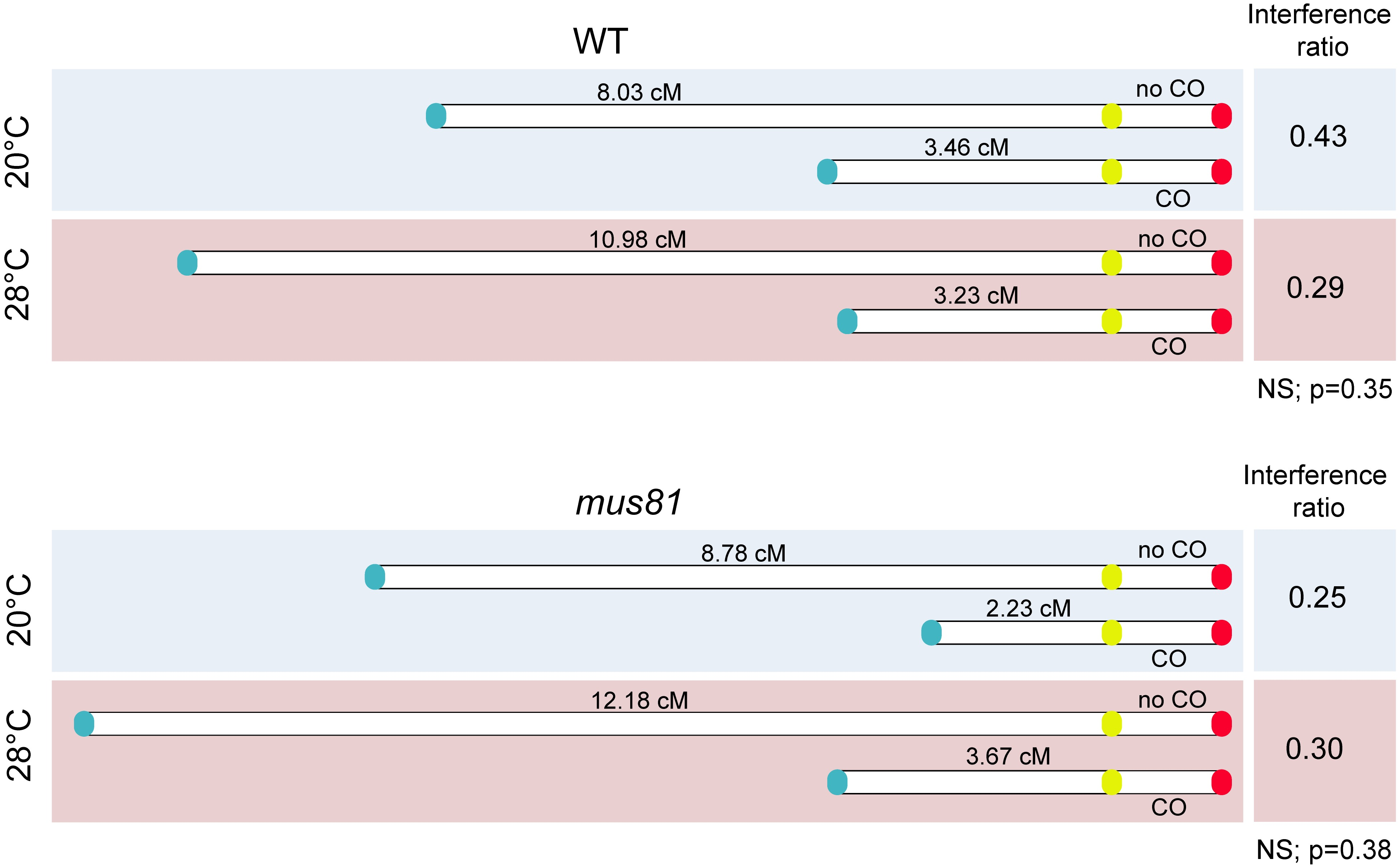

Supplement: S1 Fig — Genetic distances measured using the I5cd FTL interval in WT and mus81. Genetic distance measured in the I5c interval both with and without COs in the adjacent I5d interval. CFP, YFP and dsRED transgenes shown in blue, yellow, and red, respectively. (TIF) [file pgen.1007384.s001.tif]
