# Supplementary material for: Elevated temperature increases meiotic crossover frequency via the interfering (Type I) pathway in Arabidopsis thaliana
Source: PLoS Genet. 2018 May 17;14(5):e1007384. doi: 10.1371/journal.pgen.1007384 (PMC5976207; doi:10.1371/journal.pgen.1007384)
Supplement: S1 File — (PDF) [file pgen.1007384.s011.pdf]

File S1. Microscope settings for the Zeiss LSM 880 confocal laser scanning microscope used in three color (I5cd) experiment.

Objective
EC-Plan-Neofluor 10x/0.3
Pixel dwell 2.06
Speed 7
Pinhole 299.8

YFP (Track 2)
- 514nM laser
- Laser power: 2.0
- Data collected from 519-556 nM
- Gain (Master): 808
- Digital offset: 0
- Digital Gain: 1.0

CFP (Track 3)
- 458 nM laser
- Laser power: 8.0
- Data collected from 463-511 nM
- Gain (Master): 765
- Digital offset: 0
- Digital Gain: 3.0

dsRED (Track 3)
- 561 nM laser
- Laser power: 1.0
- Data collected from 566-700 nM
- Gain (Master): 688
- Digital offset: 0
- Digital Gain: 1.0

Brightfield (Track 3)
- Gain (Master): 178
- Digital offset: 0
- Digital Gain: 1.0
